# Sickle cell disease detection in low-resource conditions using transfer-learning and contrastive-learning coupled with XAI

**DOI:** 10.1038/s41598-026-35831-9

**Published:** 2026-01-24

**Authors:** Jay Patel, H. Muralikrishna, Krishnaraj Chadaga, Ananthakrishna Thalengala, Niranjana Sampathila

**Affiliations:** Manipal Institute of Technology, Manipal Academy of Higher Education, Manipal, India

**Keywords:** Sickle cell disease detection, Machine learning, Explainable artificial intelligence, Transfer-learning, Contrastive-learning, Good health and well-being, Sickle cell disease, Diagnostic markers, Computer science

## Abstract

Sickle cell disease (SCD) is a severe hereditary blood disorder that affects millions worldwide, necessitating early and accurate detection to improve patient outcomes. State-of-the-art approaches for automatic detection of SCD use deep learning (DL) based models, which require a large amount of training data for efficient training. However, such large training datasets are often not available, significantly limiting the efficiency of DL-based models. In this paper, we propose different approaches to address this issue. Firstly, we propose to use a transfer-learning based approach, where we use pre-trained models like ResNet-50, DenseNet-121, and EfficientNet-B0 and fine-tune them for SCD detection. To further enhance the efficiency of the models, we then propose to include contrastive-learning-based approach using triplet loss. We also use focal loss to handle class imbalance. Additionally, we integrate Explainable Artificial Intelligence (XAI) methodologies to interpret and explain the model’s predictions, ensuring transparency and trustworthiness in clinical settings. Experiments on a publicly available SCD image dataset show that models trained with transfer learning and triplet loss outperform those trained with binary cross-entropy or focal loss.

## Introduction

**Figure 1:**
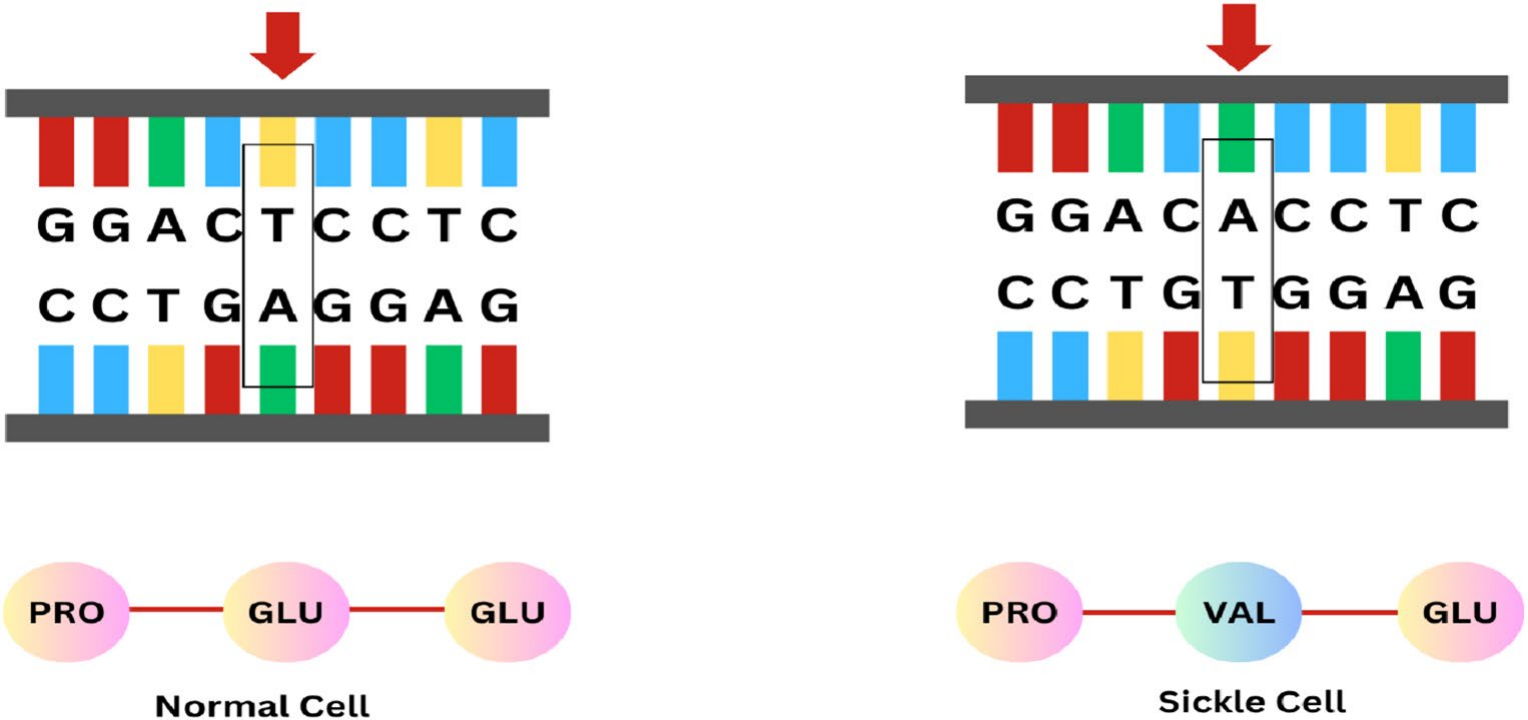
Detailed illustration of genetic mutation and hemoglobin alteration in sickle cell disease.

Sickle cell disease (SCD) is a genetic blood disorder where we find the presence of abnormal hemoglobin, known as hemoglobin S, which causes red blood cells (RBCs) to assume a rigid, sickle-like shape. These abnormal cells can lead to various health complications, including pain episodes, anemia, swelling, infections, and organ damage^1–3^. The mutation in the hemoglobin gene reduces the flexibility of RBCs due to which they get trapped in small blood vessels, leading to restricted blood flow. This restriction can cause severe pain and damage to organs and tissues. Figure 1 depicts the mutation process caused by sickle-shaped cells.

SCD is most prevalent among individuals of African, Mediterranean, Middle Eastern, and Indian ancestry, affecting millions of people worldwide. The World Health Organization (WHO) identifies SCD as a major public health concern due to its significant impact on morbidity and mortality rates^2–4^.

**Figure 2:**
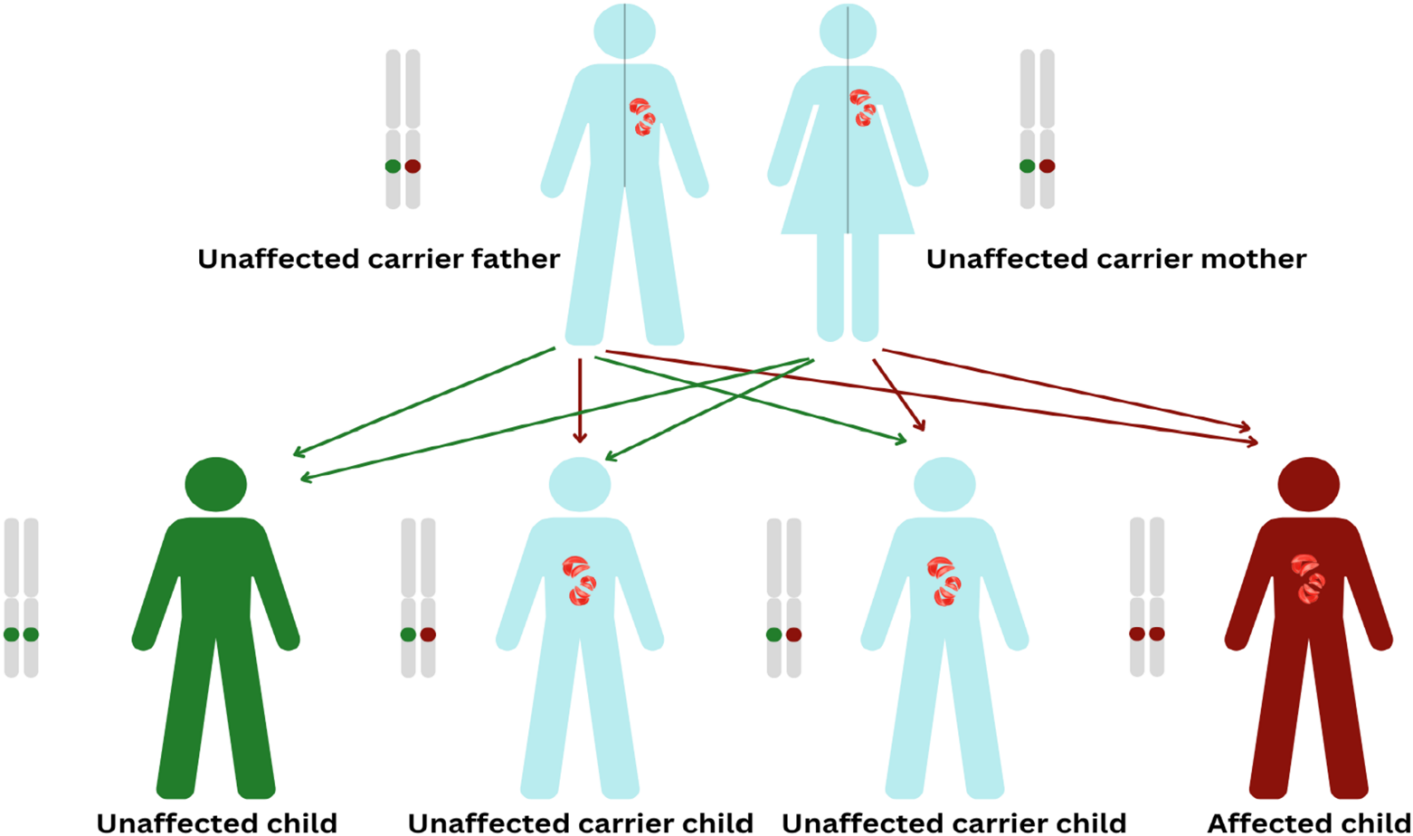
Inheritance patterns of sickle cell disease and genetic transmission scenarios from carrier parents to offspring.

WHO states that roughly 5 percent of the population carries trait genes for sickle cell disease globally. This percentage crosses the 20 percent mark in some regions, predominantly in sub-Saharan Africa, India, Saudi Arabia, and Mediterranean countries, where over 300,000 babies are born with hemoglobin disorders annually^5^. Hospitals in the USA report over 75000 hospitalizations related to SCD, costing over 300 million pounds per year. The burden of SCD is most concerning in the regions of Nigeria and the Democratic Republic of Congo where its birth rates are tremendously high^6–8^. SCD is a critical public health challenge in India. There are about 150,000-200,000 children born each year with SCD, with a devastating mortality rate ranging between 50-80 percent before they turn five years old. Even though it affects more than twenty million individuals, SCD is mostly underdiagnosed and thus silent. The nature of SCD is autosomal recessive, that is, that a person must receive two copies of the sickle cell gene, one from each parent, to develop the disease. Individuals with only one copy of the gene are carriers and normally do not show symptoms but can pass the gene to their offspring. Figure 2 shows the inheritance pattern of the SCD.

SCD is usually diagnosed by examining the blood sample under a microscope. Accurate and early identification is critical for better disease management and timely preventive care, which is closely aligned with the United Nations SDG-3 (Good Health and Well-Being). However, manual classification of sickle cells is quite challenging due to the irregular and complex shapes of the cells. This method is highly time-consuming and prone to errors, as it requires distinguishing between millions of red blood cells in a smear. Key complications include overlapping, blurry boundaries, colour complications, etc.^9–11^. Traditional diagnostic methods, such as hemoglobin electrophoresis and genetic testing, are reliable but can be costly, and require specialized equipment and expertise. Classical electrophoresis methods, such as cellulose acetate and citrate agar, are commonly used to separate and identify hemoglobin variants based on their charge differences in an electric field. Although cost-effective, this approach is labor-intensive and time-consuming, requiring two-tiered electrophoresis protocols at both alkaline and acidic pH levels. In resource-limited settings, these factors can hinder timely diagnosis and intervention. While the hemocytometer is widely used for blood cell counts, it is expensive and not readily available in many hospitals. This limitation has led to the development of computerized systems that identify anemia types using machine learning algorithms^12^.

With advancements in medical imaging technology, we now have access to high-resolution microscopic digitized images of blood samples. These images provide detailed visual information that can be leveraged by AI and ML techniques to enhance the diagnostic process. By applying AI and ML to these digitized images, we can automate the classification of sickle cells, reducing the time and effort required for diagnosis. This approach not only increases efficiency but also improves accuracy by minimizing human error. Moreover, AI-driven diagnostics can be deployed in resource-limited settings, offering a scalable solution to overcome the limitations of traditional methods. The integration of AI and ML in SCD diagnosis represents a significant step forward in making reliable and timely diagnosis accessible to a broader population.

In recent times the healthcare field has progressed by leveraging Artificial Intelligence in diagnosis and treatment of patients using their data. Deep learning methods and their multi-level representation-learning algorithms that each transform a low-level representation into a higher one have portrayed great performance and potential in AI, image processing tasks, and more^13^. Handling of complex and multi-modal data has become simpler and majorly the task of feature engineering has been shifted from humans to machines^13,14^. In addition to deep learning algorithms, XAI techniques like Grad-Cam, LIME, SHAP, Saliency Maps, etc. have significantly contributed in clarifying the outputs of their algorithms, dealing with the black-box nature of DL models and shedding light onto the neural network’s prediction process^15^.

## Literature review

Many researchers have studied SCD using deep learning algorithms and AI. In^16^, Aliyu et al. proposed a comparative analysis between Support Vector Machine (SVM) and AlexNet models, concluding that SVM outperformed AlexNet due to a limited dataset. Xu et al.^17^ demonstrated how deep convolutional neural networks (DCNNs) could process thousands of RBCs samples in deoxygenated states.To expand on the work by Xu et al.^17^, their approach involved extracting individual RBC patches from microscopic images and normalizing these patches to ensure uniformity in size. They then used DCNNs to classify RBC shapes into discocytes, echinocytes, and sickle cells, based on morphological characteristics. This method allowed for high-throughput processing of RBC samples in deoxygenated states, effectively distinguishing cell shapes associated with SCD with notable accuracy. This automated classification reduced human error and facilitated faster diagnosis in clinical settings. This approach provided good classification accuracy. Chy and Rahaman developed an automated method for detecting sickle cell anemia (SCA) using image processing on thin blood smears. The process begins by capturing blood images with a microscope-connected camera, followed by converting images to grayscale, enhancing them, and applying a median filter to reduce noise. RBCs are then segmented using a threshold and refined with morphological operations to remove unwanted objects. Features based on color, texture, and geometry are extracted, and a classifier is trained on 120 images (80 for training, 40 for testing), achieving 95% accuracy and 96.55% sensitivity^18^. Alagu S et al. used images from the erythrocyte IDB public database and developed an automation system to detect sickle cells by optimizing the InceptionV3 model. The results were improved by introducing Multi-Objective Binary Grey Wolf Optimization (MO-BGWO) alongside KNN and SVM, achieving an accuracy of 96%. Great progress has been shown by DL techniques in biomedical data classification^19^. Goswami et al.^2^ used pre-trained CNNs and used Grad-CAM based XAI for SCD, in which the models were optimized with cross-entropy classification loss. Unlike their work^2^, our primary contribution is a metric-learning pipeline that learns discriminative embeddings via triplet loss followed by classification in the learned feature space (using a simple backend classifier). This objective directly enforces small intra-class and large inter-class distances between RBC morphologies, which is particularly advantageous for SCD where sickle vs. normal cells can be visually similar and heavily confounded by staining artifacts. We further benchmark triplet loss against BCE and focal losses on both balanced and naturally imbalanced splits, showing consistent gains in generalization and stability^2^. In addition to these, there have been few other studies in the domain of SCD^11,20–22^. Table 1 shows a summary of these studies on utilizing deep neural networks (DNNs) and convolutional neural networks (CNNs) for SCD detection.

**Table 1:** Comparative analysis of sickle cell detection methods: models, datasets, performance, and future directions.

Study/author(s)	Method/model	Performance metrics	Advantages
John Paolo et al.^20^.	YOLOv3	100% accuracy in detecting sickle cells	High accuracy using YOLOv3-Tiny
Matheus et al.^22^.	Custom CNN, Bayesian Optimization	92.54% accuracy (custom CNN)	Optimized augmentation reduces overfitting
Mengjia Xu et al.^21^.	UNet	High accuracy in classifying 1,000+ RBCs	Fast, accurate RBC type classification

Apart from detection of SCD, ML-based techniques have greatly influenced automatic detection of abnormalities/complications resulting from sickle cell anemia, such as organ dysfunction. For example, Mohammed A et al.^23^ proposed a multilayer perceptron model to analyze the predictions of organ dysfunction in ICU-admitted SCD patients. A high accuracy was achieved with 96% sensitivity. This study also proved the advantage of utilizing ML in the early detection of SCD complications as it could predict organ dysfunction up to 6 hours before onset.

Despite the advances in DL/ML, automating the process of SCD detection is challenging due to factors like overlapping of cells and congestion, blurry boundaries, colour complications, etc.^13,17^. These factors create confusion to the model, by increasing inter-class similarities and intra-class variations, leading to degraded performance. The lack of sufficient training samples to train the DL models is another significant obstacle. Note that, in such situations, state-of-the-art DL-based models cannot guarantee a satisfactory performance.

## Contribution

In this paper, we propose to bridge these research gaps. Specifically, we propose using a transfer-learning-based approach, combined with contrastive learning, to handle the issue of limited training samples. We first explore the usage of state-of-the-art pre-trained DL models such as ResNet-50, DenseNet-121, and EfficientNet-B0 for SCD detection. We then apply contrastive learning using triplet loss, followed by focal loss to handle class imbalance^24,25^ on these models to improve their performance in low-resource conditions. Furthermore, the XAI techniques help in interpreting the model’s predictions. In addition to this, we also explore the benefits of using a separate back-end classifier, designed to classify the embeddings from pre-trained DL models, for SCD detection. Specifically, we use the k-nearest neighbor (KNN) classifier^26^.

The highlights of this paper are listed below:

1. Employment of a transfer-learning approach using state-of-the-art pre-trained models like ResNet-50, DenseNet-121, and EfficientNet-B0 for SCD detection in low-resource conditions, using digitized thin blood films.
2. Effective utilization of triplet loss and focal loss for SCD detection in low-resource conditions.
3. Usage of XAI for improving transparency and interpretability of the used models.

**Figure 3:**
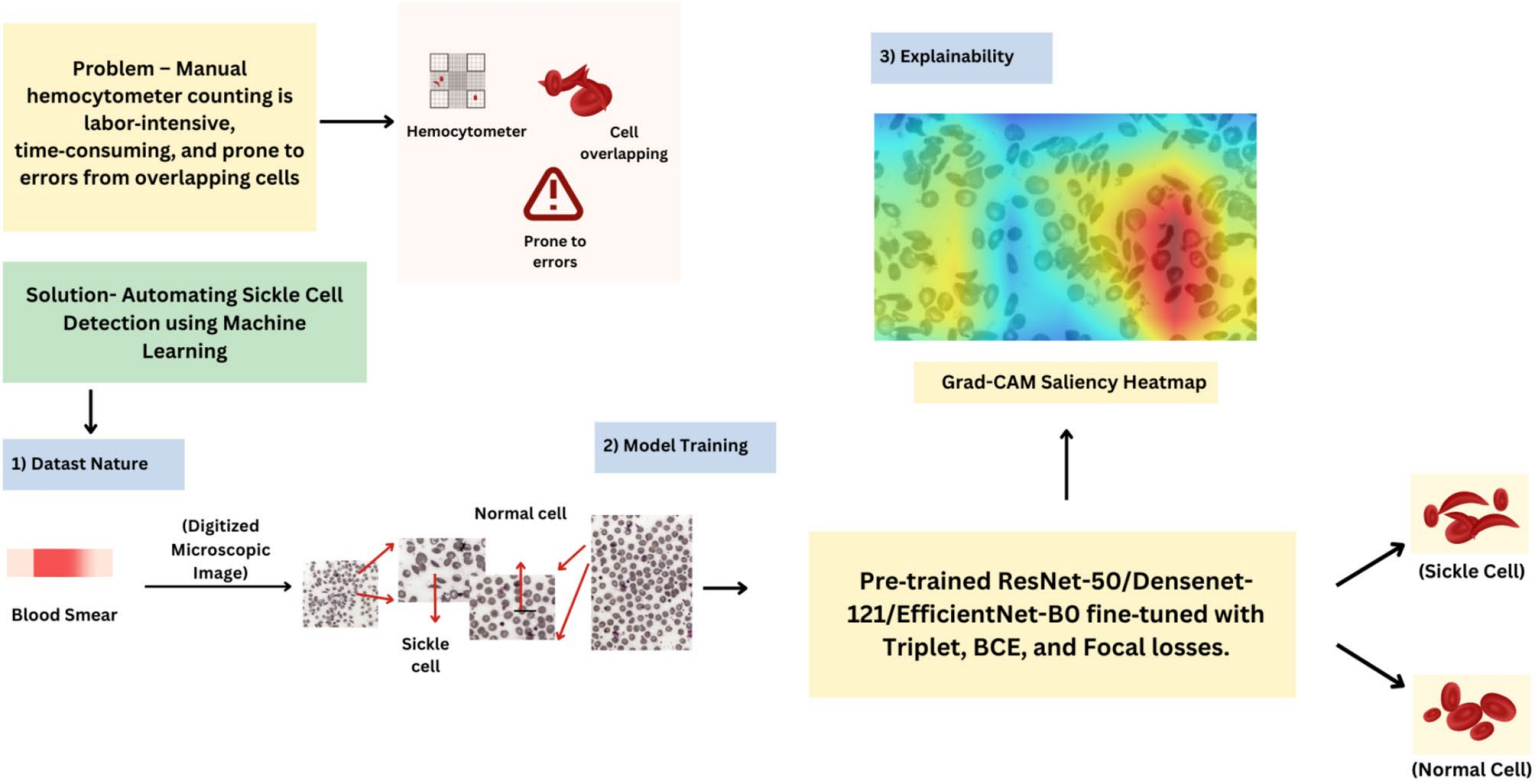
Workflow of the study.

The rest of this paper is organized as follows. In “SCD detection using transfer-learning and contrastive-learning”, we discuss the models and loss function used for SCD detection (methodology). In “Dataset” we discuss the datasets used in this work. The details about experiments are given in “Results”, followed by discussion in “Experiments with unbalanced training dataset”. The workflow follwed in this study is described in Fig. 3.

## SCD detection using transfer-learning and contrastive-learning

**Figure 4:**
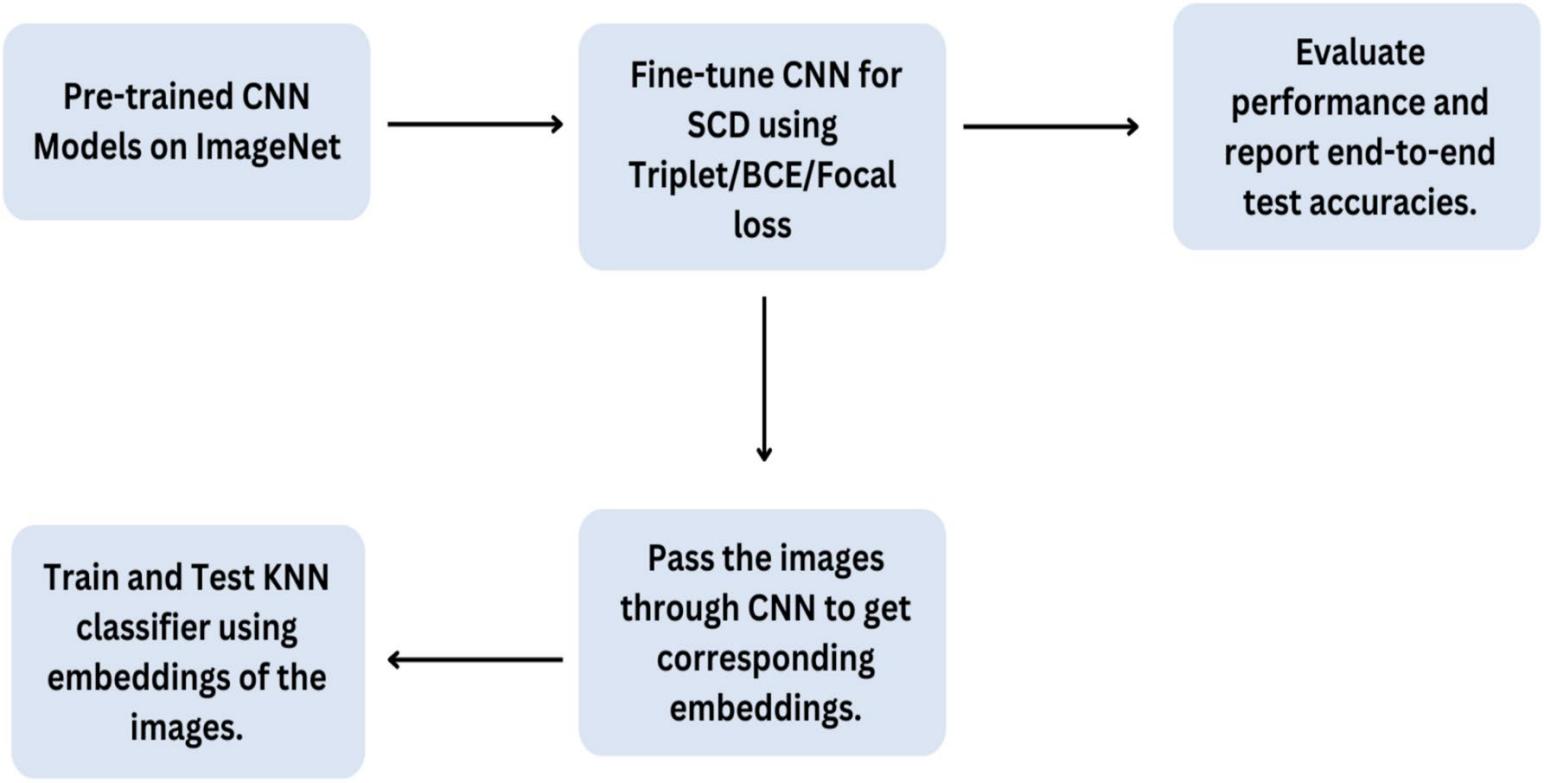
Transfer learning workflow for sickle cell disease classification.

CNNs have shown great dominance in pattern recognition with huge digitized image data. This ability of CNNs has helped the medical field simplify the disease identification process. ResNet-50, DenseNet-121, and EfficientNet-B0 are some widely used CNNs for solving computer vision problem statements. Each model has a unique architecture that helps extract meaningful features from images^27–30^. In our work we fine-tune the CNNs to specific SCD dataset to adapt these models. Subsequently, these refined models are used to extract feature embeddings, which are then used to train a separate K-nearest neighbor (KNN) classifier, effectively combining the robust feature extraction capabilities of deep learning with the simplicity and effectiveness of a traditional machine learning classifier for the final classification of SCD. Figure  4 below illustrates this process.

### ResNet-50

**Figure 5:**
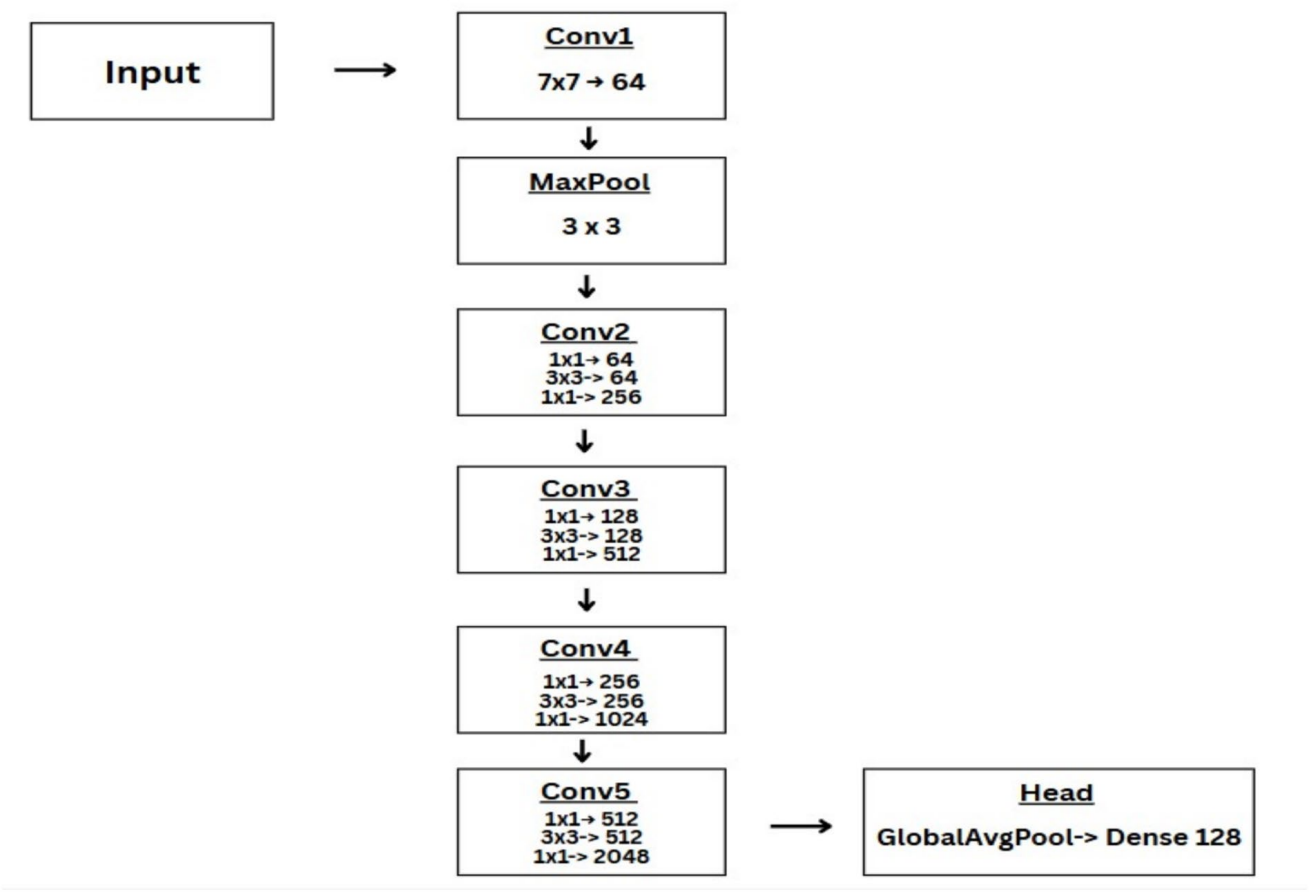
ResNet-50 architecture overview.

ResNet-50 is a deep CNN well-trained on the Imagenet dataset. The input layer accepts an image of size 224x224 which is then passed to the first convolutional layer (Conv1x) with 64 filters and 7x7 kernel size, followed by a 3x3 MaxPooling Layer with a stride of 2. Next comes the remaining residual blocks Conv2x, Conv3x, Conv4x, and Conv5x with Con2x having 64-64-256 filters after which they are multiplied by a factor of 2 for each layer. A Global Average Pooling layer is applied before the fully connected Dense Layer. In our work, we train this network using three different loss functions i.e., triplet loss, binary cross entropy (BCE) and focal loss. In the case of triplet loss, we use Relu activation for the last layer, and we use sigmoid activation in the case of BCE and focal loss^31,32^. Figure 5 shows the ResNet-50 architecture diagram with the key hyperparameters used in our implementation.

### DenseNet-121

**Figure 6:**
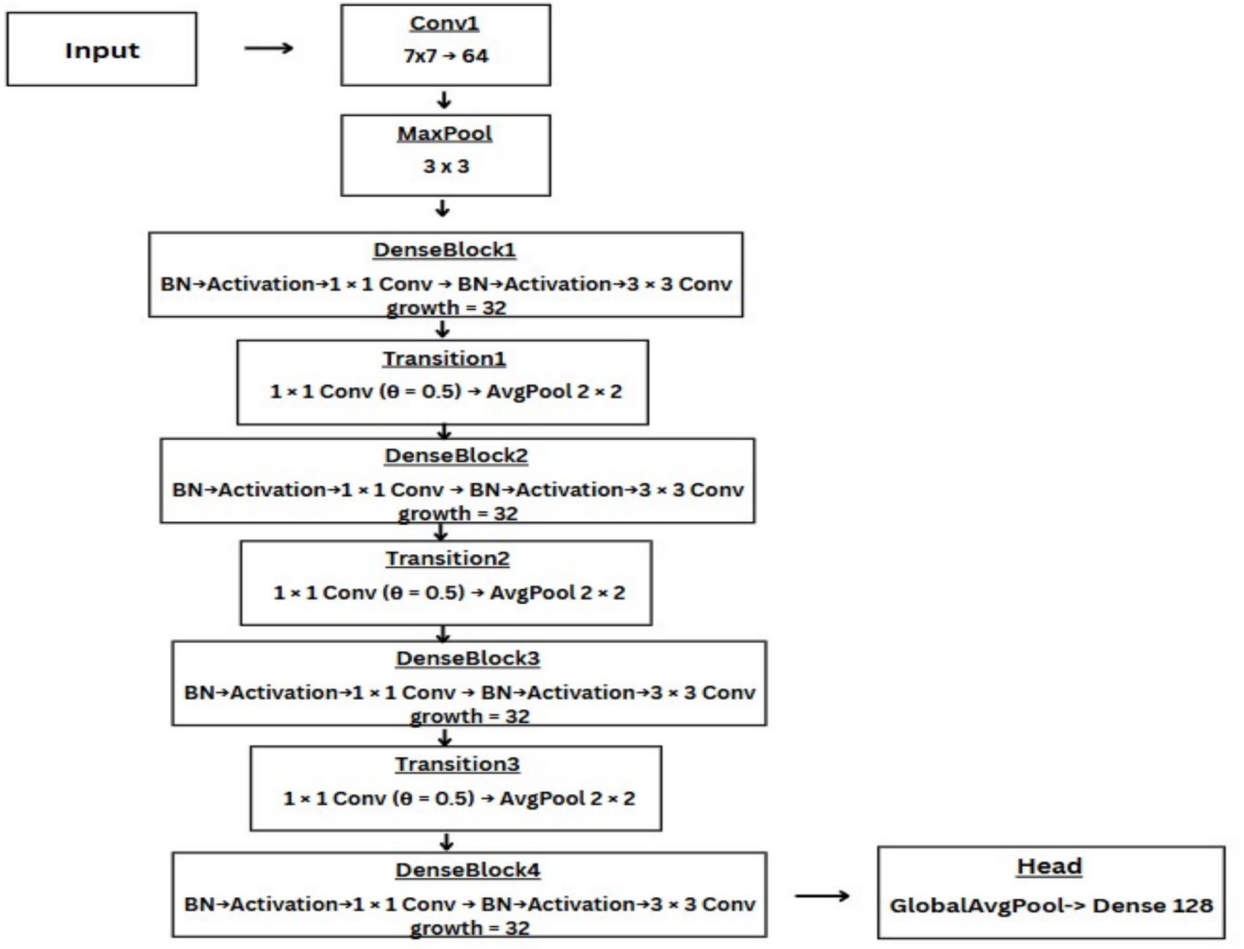
DenseNet-121 architecture overview.

DenseNet-121 is a unique CNN that is trained using images from the Imagenet database. DenseNet-121 architecture comprises a dense connection of all the layers, that is, every layer makes use of the inputs from all the previous layers to draw a feature map. Hence the output of the $m$th layer will look like this:

1 $$\begin{aligned} x^{(m)} = H^{(m)} \left( [x^{(0)}, x^{(1)}, x^{(2)}, \ldots , x^{(m-1)}] \right) \end{aligned}$$

where $[x^{(0)}, x^{(1)}, \ldots , x^{(m-1)}]$ is a collective representation of the previously extracted feature maps and $H^{(m)}$ represents the $m^{th}$ layer. Hence, in DenseNet-121 architecture the features are reused. The Initial layer again consists of 64 filters, 7x7 kernel size followed by a 3x3 MaxPooling Layer with 2 strides. There are 4 Dense blocks (1,2,3,4), each having a transition layer in between. DenseNet has been successfully used for different applications in the past^33,34^. Similar to the ResNet-50 model, we use three different loss functions to train the model. Figure 6 shows the DenseNet-121 architecture diagram with the key hyperparameters used in our implementation.

### EfficientNet-B0

**Figure 7:**
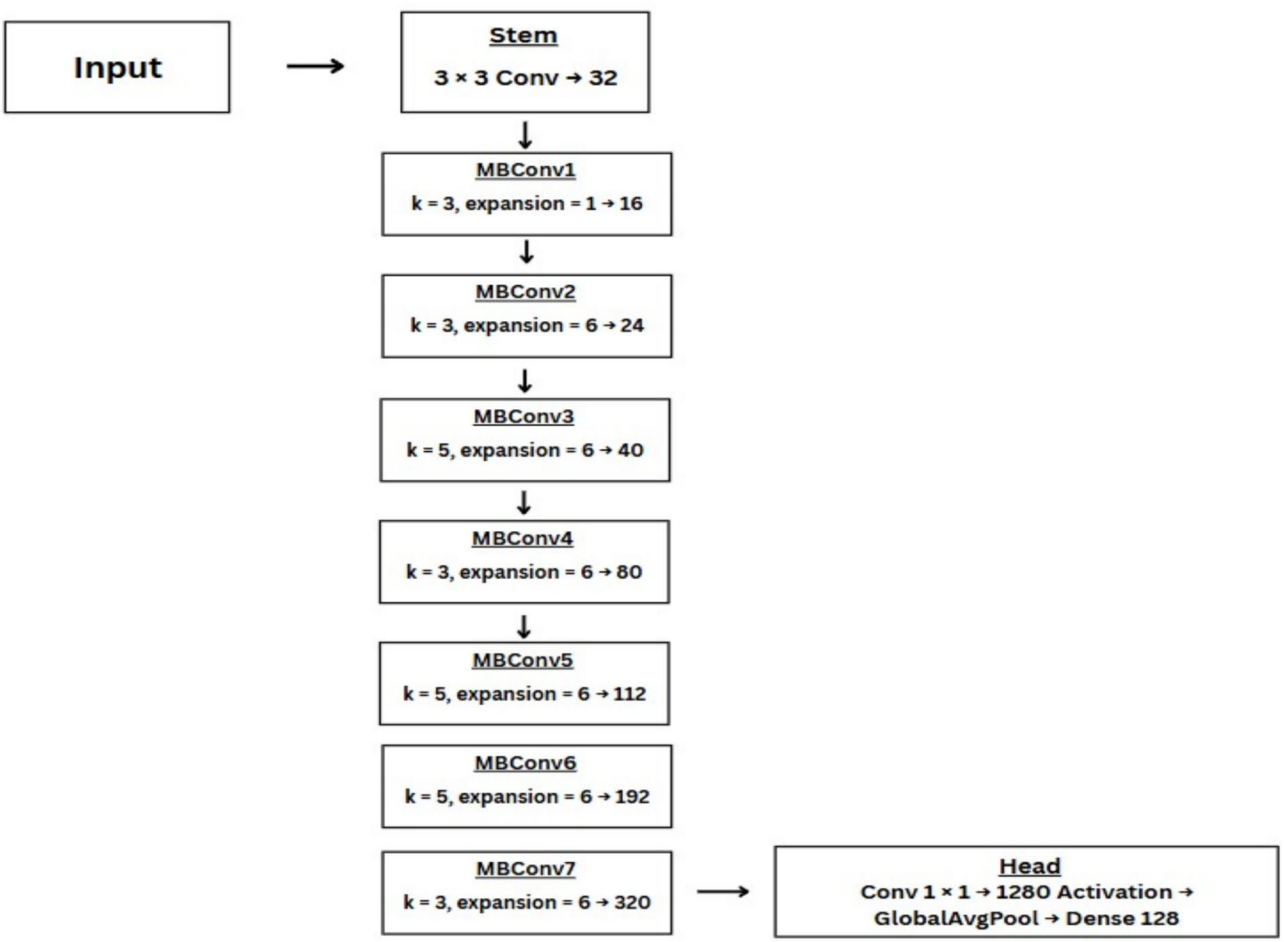
EfficientNet-B0 architecture overview.

EfficientNet-B0, a newly emerged CNN model for image classification, has a powerful scaling technique that it utilizes by applying compound coefficients to scale up width, depth, and resolution dimensions uniformly. The input layer takes a 224x224 input and passes it onto Conv2DLayer of kernel size 3x3 and 32 filters. There are multiple convolution layers with either 3x3 or 5x5 size which reduce resolution while increasing the width. The filters keep on incrementing from 32 onwards to 1280. The ‘MBConv’Efficient Blocks can be customized based on the type of dataset^35–37^. Figure 7 shows the EfficientNet-B0 architecture diagram with the key hyperparameters used in our implementation.

### Lightweight baseline (MobileNetV2)

To provide a capacity-matched baseline suited for small datasets, we additionally evaluate MobileNetV2 ($\approx$3.5M parameters) both with ImageNet pretraining and from scratch (weights=None). We keep the same input resolution, augmentations, training schedule (epochs, batch size, learning rate), and loss functions (triplet, BCE, focal) as the larger backbones to enable a fair comparison

## Analysis of loss functions

DL neural networks determine the function that correctly maps the input to the output. We try to obtain this function by minimizing empirical risk on the training sample using a specific loss function. By making a differentiation between the predicted values and actual target values, these functions give us insights on the strength of the models in predicting the expected outcomes. Hence, a model can be optimized by achieving a minima of these loss functions^38,39^.

In this paper, we explored the usage of three different loss functions- triplet loss, BCE loss, and focal loss for efficient SCD detection^25,40,41^. Each model has been executed individually with each of these loss functions in order to evaluate their performance. This also helps us understand how a specific model architecture behaves when different loss functions are used.

### Triplet loss

Triplet loss is widely used for image classification tasks. Triplet loss is particularly beneficial in low-resource conditions as it enables discriminative learning of representations without needing a large number of labeled samples. Triplet loss is a supervised metric-learning objective that requires ground-truth class/identity labels to form valid (anchor, positive-same class; negative-different class) triplets; without these labels, triplet sampling is ill-posed and the objective can degenerate. Considering an anchor image $x_a$ and a positive image $x_p$ belonging to the same class $y_a$ and a negative image $x_n$ belonging to another class $y_n$, this loss function validates that the projection of the positive point and the anchor point are closer to each other in the embedding space as compared to the projection of the negative point by at least a margin $m$. In our case, each image is converted into an embedding using the neural network. Triplet loss is mathematically represented as:

2 $$\begin{aligned} L(a, p, n) = \max (0, d(a, p) - d(a, n) + m) \end{aligned}$$

where $a$ is the embedding of the anchor, $p$ is the embedding of positive sample, $n$ is the negative sample, $d$ is the distance metric, and $m$ is the margin. This loss function helps the model to minimise the distance between the embeddings of the same class. Triplet Loss enforces hinge-based margin in the learned embedding manifold and maximizes inter-class separation (sickle vs. normal) while minimizing intra-class dispersion, yielding tighter clusters for each cell type. While triplet loss is not a classification loss in the strict sense, it is used here as a metric learning objective. Specifically, the Neural Net backbone is trained to produce a 128-dimensional embedding space in which sickle and normal red blood cells (RBCs) are well separated: embeddings from the same class are pulled closer together, and embeddings from different classes are pushed apart by at least the margin. To convert these embeddings into class predictions, we used a centroid based approach where class label is obtained by computing L2 distance between the test embedding and the class centroids.

### Binary cross entropy (BCE) loss

As the name suggests, BCE loss helps the model to learn binary classification by differentiating between the predicted and actual probabilities. This loss increases if the predicted probability of the image classification strays away from the actual class^42^. The mathematical representation of BCE is as follows:

3 $$\begin{aligned} L(y, y_p) = -[y \log (y_p) + (1-y) \log (1-y_p)] \end{aligned}$$

where $y$ is the actual label and $y_p$ is the predicted label. Note that, a poor model will predict the probability of a positive class (with true label as 1) close to 0, and will predict the probability of a negative class (with true label as 0) close to 1, leading to an increase in the loss value. The mapping of the input values to the [0, 1] range is performed by the Sigmoid function given by:

4 $$\begin{aligned} \sigma (x) = \frac{1}{1 + e^{-x}} \end{aligned}$$

### Focal loss

Focal Loss extends BCE to address class imbalance by reducing the loss for well-classified (easy) examples so the model focuses more on hard-to-classify (misclassified) examples^43^. The starting point of this function is the same as that of BCE:

5 $$\begin{aligned} L_{CE}(y, y_p) = -[y \log (y_p) + (1-y) \log (1-y_p)] \end{aligned}$$

where $y$ is the actual label and $y_p$ is the predicted label. After this step, a factor of $\alpha$ is introduced to balance the positive/negative samples:

6 $$\begin{aligned} L_{\alpha }(y, y_p) = - \alpha [y \log (y_p) + (1-y) \log (1-y_p)] \end{aligned}$$

Finally a factor of $(1 - y)^r$ is introduced with $r$ as the tunable focusing parameter to ensure the contributions of hard-to-classify samples in the loss function:

7 $$\begin{aligned} L_{FL}(y, y_p) = - \alpha (1 - y)^r [y \log (y_p) + (1-y) \log (1-y_p)] \end{aligned}$$

Table 2 gives a summary of these loss functions.

**Table 2:** Different loss functions used in this work for SCD detection.

**Loss Function**	**Applications**
Triplet Loss	Uses two same-class images (anchor, positive) and a different-class image (negative).
Binary Cross-Entropy	Labels: 0 for normal, 1 for sickle. Minimizes prediction error.
Focal Loss	Similar to BCE but focuses on hard-to-classify examples.

## Model training

This study focuses on designing deep learning models optimized for robust image classification even with a dataset that is small in quantity. The obtained dataset was split into approximately 80-20 ratio for training and validation respectively, after which a separate test dataset, containing 100 images each in the sickle and normal category, was utilized for testing the trained model and implementing Grad-CAM. In this experiment, we have used transfer learning, where we used pre-trained CNNs (trained on Imagenet dataset) and finetuned them to classify sickle cell images^44^.

**Figure 8:**
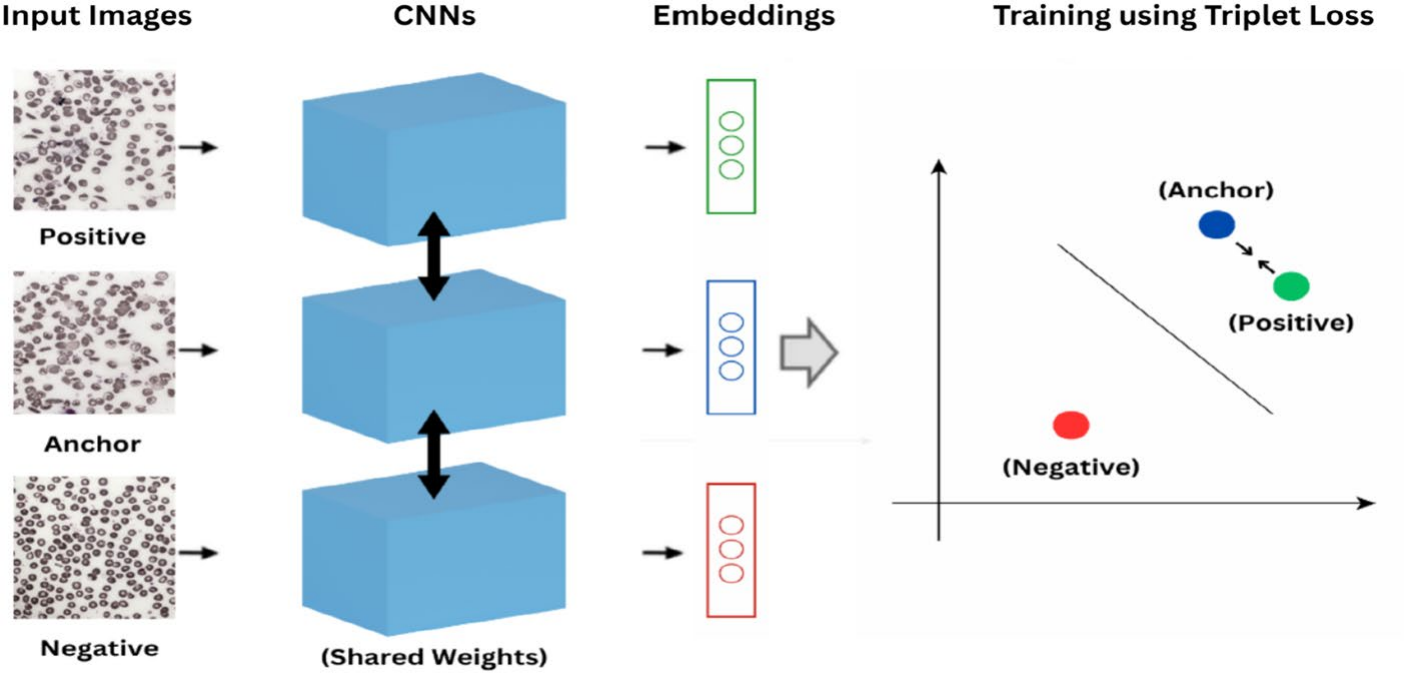
Triplet loss training for image embedding.

In our approach, we finetuned the model using triplet loss, BCE loss, and focal loss, without freezing the initial layers. New layers such as Global Average Pooling, Dropout, and Batch Normalization were added on top of the pre-trained models and each layer (including the early layers) underwent updates during the training. This allowed the final weights of these learned layers to specifically align with sickle cell characteristics enabling better classification. Figure 8 illustrates the triplet loss based training. The discriminative abilities of the triplet models were enhanced by employing a‘Semi-Hard Triplet Mining’strategy^45,46^. Using this strategy, training triplets were generated, which were challenging but attainable, to distinguish the negative samples from the positive ones, in contrast to the very easy/difficult negatives. This ensured a faster convergence, enabling the model to learn more discriminative features. To mathematically represent this strategy, we begin by calculating the squared Euclidean distance metrics as:

8 $$\begin{aligned} & d(A, P) = \Vert E_A - E_P \Vert ^2 \end{aligned}$$

9 $$\begin{aligned} & d(A, N) = \Vert E_A - E_N \Vert ^2 \end{aligned}$$

where $A$, $P$, and $N$ are the anchor, positive and negative images respectively. According to the semi-hard condition, a triplet ($A, P, N$) can be formed if:

10 $$\begin{aligned} d(A, P)< d(A, N) < d(A, P) + \alpha \end{aligned}$$

where $\alpha$ is the margin parameter.

The initial learning rate indicates the extent to which the model learns to capture the relevant features and adjust its parameters while moving towards a lower value of the loss function. A high value for learning rate can lead to overshooting of the minimum, whereas a low value can slow down the convergence process. We have employed a static learning rate of 0.0001 in our models operating on triplet and BCE loss whereas a ‘ReduceLRonPlateau’callback has been utilized in focal Loss models whose initial learning rates are 0.0001. However, they decay by a factor of 0.2 for zero improvement in the validation loss for 3 consecutive epochs^47^.

The batch size was set to 16 in the case of triplet loss and 32 for BCE loss and focal loss. Adam optimizer was used as it ensures faster convergence since it updates the learning rate for each weight dynamically. The experiment was conducted using Google Collab Pro’s T4 GPU.

## XAI using grad-CAM

The complex architectures of deep learning models can pose interpretability challenges when it comes to the decisions that they make. Certain deep learning neural networks are “black box”models as they hinder transparency. A model may achieve high accuracy but it need not guarantee generalizability, as it may have done so by exploiting irrelevant features, causing unreliable predictions on test data. In this research, we have used Grad-CAM for simplifying interpretability. By using the gradients of the class score of the final convolutional layer of the neural network, it extracts saliency maps highlighting the regions in an image contributing the most to the model’s decision.

Mathematically, given a target class score $y^c$ and the activations $A^k_{i,j}$ of the $k$-th feature map at spatial location $(i,j)$, Grad-CAM proceeds as follows:

1. We calculate the gradient of the class score for each activation:
  11 $$\begin{aligned} \frac{\partial y^c}{\partial A^k_{i,j}} \end{aligned}$$
2. Obtain the channel-wise importance weights by global average pooling those gradients:
  12 $$\begin{aligned} \alpha ^c_k \;=\; \frac{1}{H \times W} \sum _{i=1}^{H} \sum _{j=1}^{W} \frac{\partial y^c}{\partial A^k_{i,j}} \end{aligned}$$
3. Combine the feature maps with these weights and apply a ReLU to keep only the positive influences:
  13 $$\begin{aligned} L_{\mathrm {Grad\text {-}CAM}}^c(i,j) = \textrm{ReLU}\!\Bigl (\sum _{k} \alpha ^c_k\,A^k_{i,j}\Bigr ) \end{aligned}$$
4. Upsample $L_{\mathrm {Grad\text {-}CAM}}^c$ to the input resolution and overlay it on the original image to produce the final heatmap.

Here, $A^k_{i,j}$ is the activation at a location (*i*, *j*) of the *k*-th feature map in the final convolutional layer of size $H\times W$, $y^c$ is the score (pre-softmax) for class *c*, $\alpha ^c_k$ is the global average-pooled importance weight for feature map *k* and class *c*, and $\operatorname {ReLU}(\cdot )$ zeroes out any negative contributions. By enabling the visualization of these activation maps, we can understand how each neuron contributes in the classification decision. The balance between high-level semantic and spatial information in the last convolutional layers allows Grad-CAM to pinpoint specific regions in an image that correspond to the features the model has learned, providing a visual confirmation that the model is taking decisions using relevant features and enhancing interpretability. This acts as a communication bridge between deep learning researchers and medical practitioners, fostering trust in DL models for sensitive tasks such as medical image analysis and classification.^48,49^.

## Separate back-end classifier

In this case, we use the DL models (which are already trained for SCD detection) as feature extractors and use a separate machine-learning-based back-end classifier to classify them. Specifically, we use a KNN classifier in this work. We first train the DL models (ResNet-50, DenseNet-121 and EfficientNet-B0) for the SCD detection, and report the corresponding results. We call them end-to-end models, as they directly give the classification prediction for the given input image. Following this, we use these models as feature extractors and feed the features to the back-end KNN classifier. Such a combination of deep learning and machine learning models enables us to utilize the strengths of both approaches^46^.

Formally, let

$$\begin{aligned} \phi _\theta : \mathbb {R}^{224\times 224\times 3}\;\rightarrow \;\mathbb {R}^{128} \end{aligned}$$

be the pretrained model used as embedding extractor. For each training image $x_i$ with label $y_i\in \{0,1\}$, we compute its feature vector (embedding)

14 $$\begin{aligned} z_i \;=\;\phi _\theta (x_i)\,\in \,\mathbb {R}^{128} \end{aligned}$$

At inference time, given a new image $x^*$, we similarly extract

15 $$\begin{aligned} z^* \;=\;\phi _\theta (x^*). \end{aligned}$$

We then compute the Euclidean distance between $z^*$ and each training embedding $z_i$:

16 $$\begin{aligned} d(z^*,z_i) \;=\;\Vert \,z^* - z_i\Vert _2 \;=\;\sqrt{\sum _{j=1}^{128} \bigl (z^*_j - z_{i,j}\bigr )^2}. \end{aligned}$$

Selecting the $k$ smallest distances defines the neighborhood

$$\begin{aligned} \mathcal {N}_k(x^*) \;=\;\arg \min _{\,i}^{\,k}\;d(z^*,z_i), \end{aligned}$$

and the predicted class $\hat{y}$ is given by majority vote among those neighbors:

17 $$\begin{aligned} \hat{y} \;=\;\underset{c\in \{0,1\}}{\arg \max }\; \sum _{i\in \mathcal {N}_k(x^*)} \textbf{1}\{y_i = c\}. \end{aligned}$$

## Evaluation metrics

We use accuracy, Recall, Precision and F1-score to evaluate the performances. The accuracy of a model demonstrates how well it has classified all the correct samples among the total number of samples. The precision value shows how accurately the model has classified all the positive samples. Recall or sensitivity is a metric that measures the number of true positives predicted out of the total actual positives. F1 score is computed as harmonic mean of Recall and Precision^50^.

## Dataset

**Figure 9:**
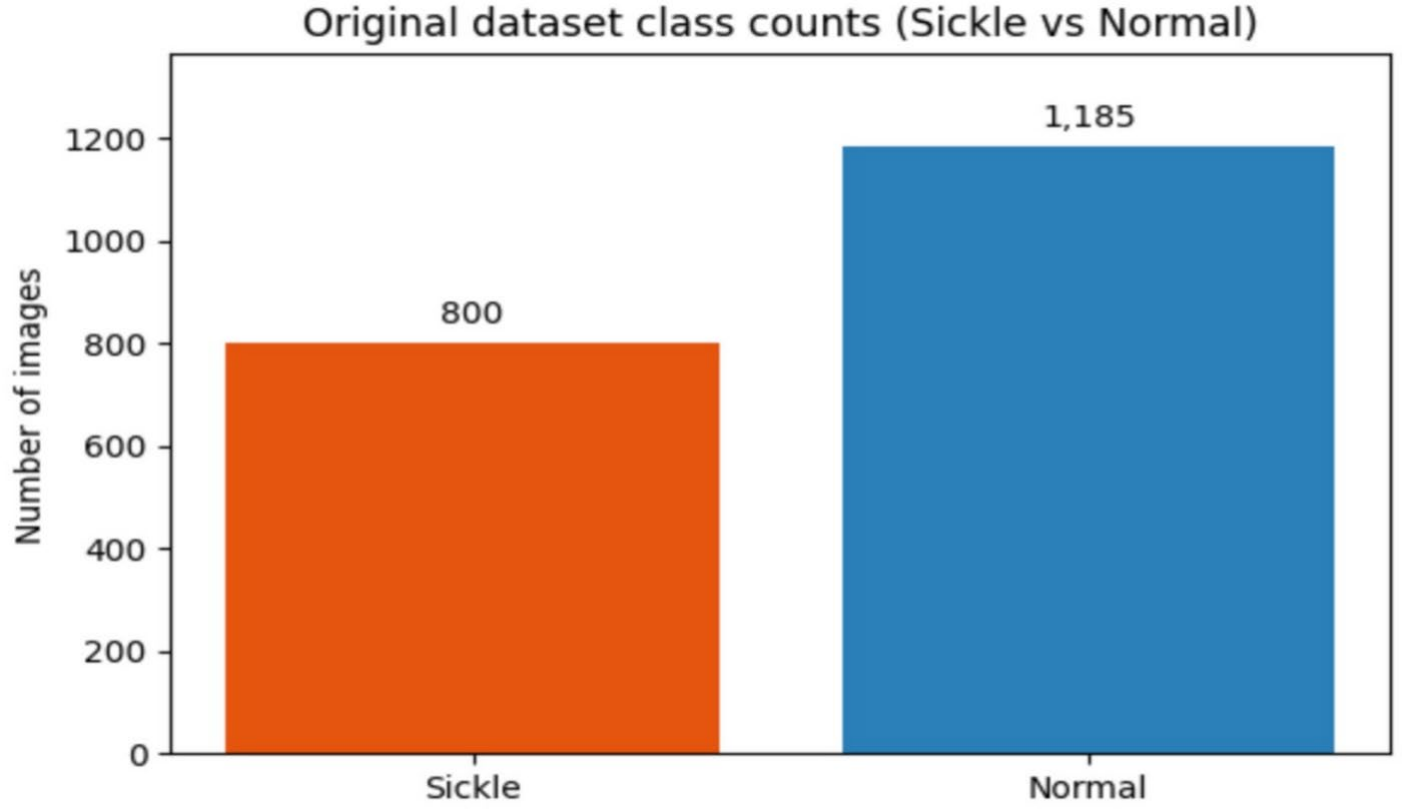
Original dataset with number of samples in each class.

**Figure 10:**
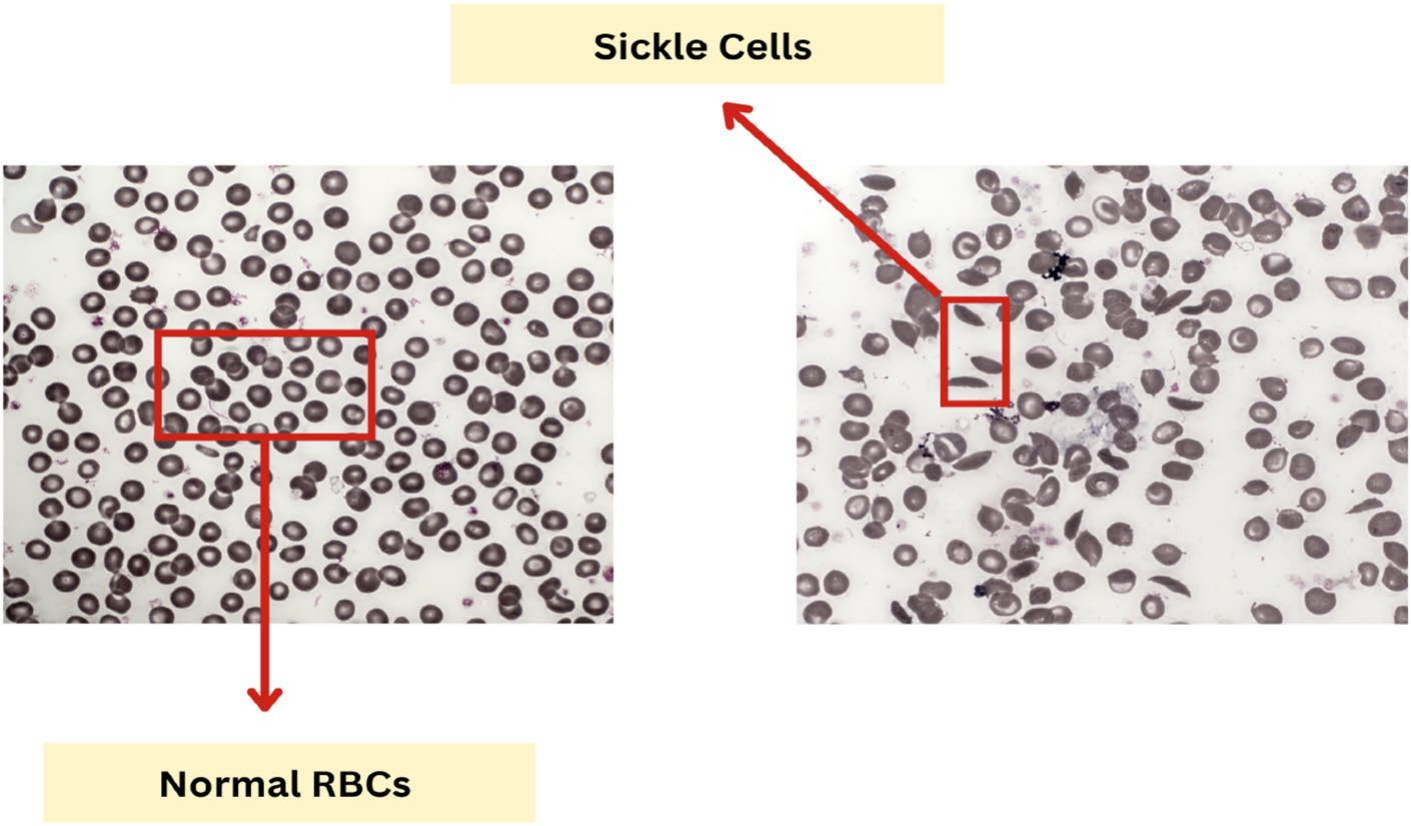
Normal cells and sickle cells images from the dataset used.

We have utilized the data from a publicly available dataset^51^. This dataset allows us to automate image analysis using deep learning algorithms to predict sickle cells. An objective lens of 1.4 numerical aperture (NA) was utilized to capture about 1985 images at 100x magnification equipped on a camera that was capable of capturing coloured images and a motorized X-Y stage for precise sample positioning. For each thick blood film sample, 100 non-overlapping fields of view (FoVs) were randomly sampled, each covering 166 $\mu$m $\times$ 142 $\mu$m area. 10 to 20 FoVs having 1500-4000 RBCs, were captured manually in blood film smears. Due to the limited depth of field from the high numerical aperture objective lens, a z-stack focal series of 14 planes, spaced 0.5 $\mu$m apart, was collected to encompass the thickness of the blood film (approximately 3 to 6 $\mu$m) for each FoV. These z-stacks were then merged into a single plane using a Wavelet-based Extended Depth of Field (EDoF) approach.^52^. Out of 1985 images, we have about 800 as sickle cells and 1185 as non-sickle cells, as displayed in Fig. 9. Figure 10 provides examples of normal and sickle cell images from this dataset, illustrating the differences in cell morphology that our models were trained to identify.

## Dataset preparation

The dataset was further modified by manual inspection so that it only contained relevant and high-quality blood smear images. After this step, to further create a balanced dataset, a Python loop was run to reduce the dataset size by choosing only 350 images in each class, making sure that the models were robust enough even for a small dataset. The first metric was brightness for which a function was created that converted an image to the HSV color space and calculated the average value of the V (brightness) channel. To further estimate a zoom level, a function was defined that converted an image to grayscale, applied a Gaussian blur, and then performed thresholding to create a binary image. Contours in the binary were found, and the area of the largest contour was returned. This area was used as a proxy for the zoom level. This loop iterated over all the images, after which the brightness and zoom-level scores were normalized between 0 and 1 using MinMaxScaler. Using a weighted sum, these scores were combined into a single score, which was finally used to arrange the top 350 images in each class. This same methodology was applied to the images of each class in the test folder too, reducing the test images to 100 in each category. This filtering approach was selected because brightness and zoom-level are two of the most critical factors influencing red blood cell (RBC) visibility and morphology in microscopic images. Brightness ensures that the illumination is sufficient for automated feature extraction without under- or overexposure, while zoom-level (approximated by largest contour area) reflects whether cells are captured at an appropriate magnification for consistent morphological comparison. We adopted these simple, computationally efficient heuristics to enforce minimum quality standards in a reproducible manner without introducing subjectivity.

## Results

**Figure 11:**
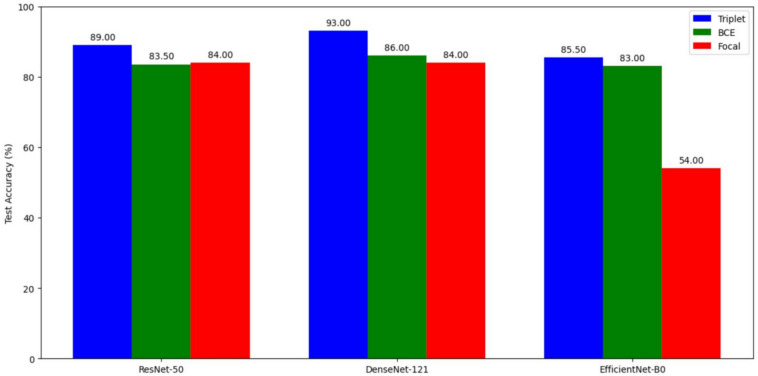
End-to-end test accuracies.

This section presents the results of the proposed SCD detection. We first evaluated the performance of various deep learning models in end-to-end mode for SCD detection. Figure 11 displays the performance (in accuracy) of every model-loss combination. ResNet-50, DenseNet-121, and EfficientNet-B0 are the three models that have been employed, which are trained using three different losses: triplet, BCE, and focal. From the results given in Fig. 11, it can be seen that the DenseNet-121 emerged as the top performer with triplet loss, with an accuracy of 93%, followed closely by ResNet-50 and EfficientNet-B0.

Note that, the choice of loss function significantly impacted the performance of all the models. Triplet loss yielded the best results as it effectively handles intra-class variability and inter-class similarity, followed by BCE loss which optimizes the predicted probabilities directly with the true binary labels, and then focal loss. Unlike standard cross-entropy, which only cares about the correct label for each example, triplet (or contrastive) loss directly shapes the embedding space by *minimizing* distances between an anchor and a positive (same class) while *maximizing* distances to a negative (other class). In general, we found that models trained with triplet loss achieved higher validation and test accuracy than their cross-entropy or focal-loss counterparts, highlighting its suitability for scarce-data medical imaging tasks.

A fair consistency was seen in every model’s performance with binary cross-entropy loss too, as it optimizes the predicted probabilities directly with the true binary labels which are notable in medical image classification tasks. DenseNet-121 and ResNet-50 showed reasonable metrics with focal loss as well; however, EfficientNet-B0 significantly under-performed with this loss function, yielding a test accuracy of just 54%. This may be because of the following reasons. The EfficientNet-B0 uses a‘compound scaling’method that simultaneously scales width, depth, and resolution, making it ultra-sensitive while calculating the loss function at each step^53^. This can intensify the instability caused by the dynamic weighting mechanism of focal loss, leading to convergence problems. In addition to this, the architecture of EfficientNet-B0 is designed to achieve computational efficiency, which may adversely amplify the overfitting effects on hard examples emphasized by focal loss. In comparison, ResNet-50 and DenseNet-121 have a straightforward architecture containing residual and dense connections, which provide better gradient flow with every dynamic adjustment, resulting in more stability.

**Table 3:** Results obtained using a KNN back-end classifier on balanced training dataset.

Network	Loss	Epoch	Batch size	Learn rate	Validation accuracy	Test accuracy	Precision	Recall	F-1 Score
ResNet-50	Triplet	30	32	0.0001	0.970	0.895	0.915	0.870	0.892
DenseNet-121	Triplet	30	32	0.0001	0.991	0.935	0.948	0.920	0.934
EfficientNet-B0	Triplet	30	32	0.0001	0.953	0.860	0.815	0.930	0.869
ResNet-50	BCE	30	16	0.0001	0.941	0.850	0.836	0.870	0.852
DenseNet-121	BCE	30	16	0.0001	0.971	0.880	0.895	0.860	0.877
EfficientNet-B0	BCE	30	16	0.0001	0.920	0.840	0.877	0.790	0.831
ResNet-50	Focal	30	32	0.0001	0.891	0.850	0.836	0.870	0.852
DenseNet-121	Focal	30	32	0.0001	0.915	0.855	0.858	0.850	0.854
EfficientNet-B0	Focal	30	32	0.0001	0.741	0.565	0.560	0.600	0.579

Following this, we experimented by using a separate back-end classifier, KNN, to see if it could improve the results. In each case, the features/embeddings obtained from the deep learning models, for all training images, are used to train the KNN classifier following which the features obtained from test images are used for testing the classifier. The value of *K* is empirically set as 5. Table 3 illustrates the results obtained for the KNN Classifier. As observed, KNN classifier coupled with DenseNet-121 model trained using triplet Loss, outperformed every model, achieving the highest test accuracy of 93.50. This makes it the most effective combination for sickle cell identification in this study. KNN coupled with ResNet-50 also produced competitive results with triplet Loss, with an accuracy of 89.50 percent, followed by EfficientNet-B0 with an accuracy of 86%. This makes EfficientNet-B0 based system as the least-performing model for sickle cell classification in our experiments.

**Figure 12:**
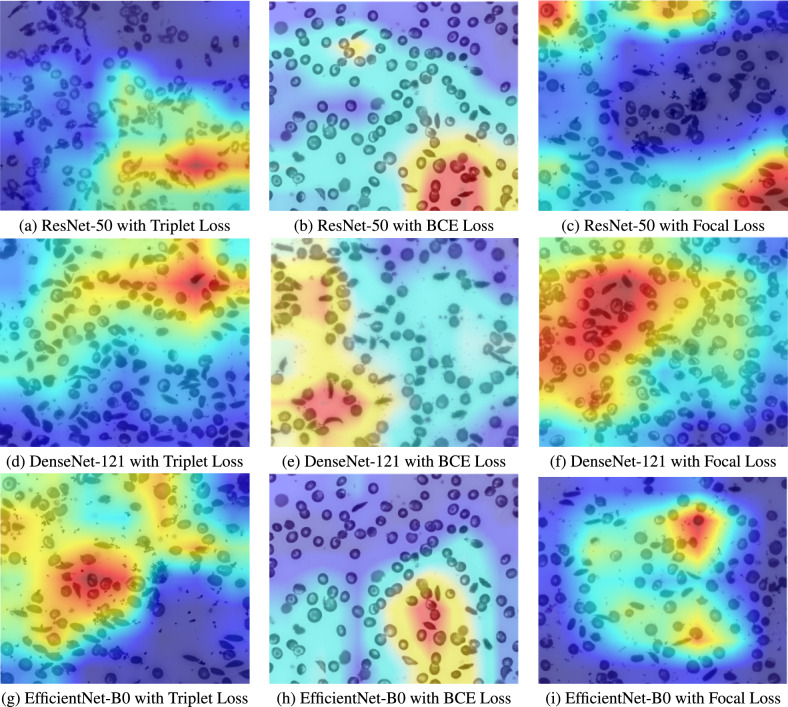
Heatmaps generated by grad-CAM for different model-loss combinations.

Next, we use Grad-CAM to improve the interpretability of our DL models. This technique generates heatmaps using gradients from the final convolutional layer so that salient pixels on it can be determined. Visualized areas on these maps represent those sections in a given input space that have been important to make decisions about whether an image belongs to the sickle class or not. Figure 12 below demonstrates the heatmaps generated by applying XAI on each model-loss combination. It is observed that the regions having a presence of sickle-shaped cells have higher heatmap values, indicating that the model has learned the correct features for predicting sickle cells in most of the cases.

**Figure 13:**
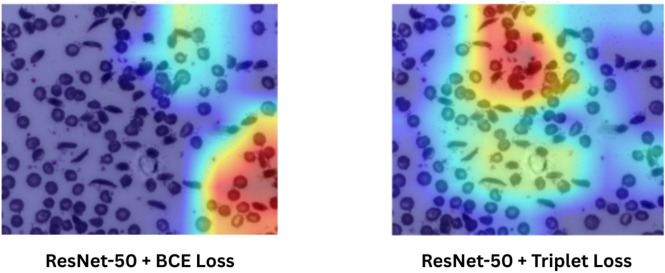
Grad-CAM comparison between ResNet-50 trained with Triplet Loss and with BCE Loss on the same test image.

Figure 13 helps us to compare the effectiveness of triplet loss and BCE loss, where same test image has been used to obtain Grad-CAM images from ResNet-50, trained respectively using BCE loss and triplet loss. It can be seen that, in case of triplet loss, the Grad-CAM heatmap cleanly highlights the curved, sickle-shaped cells, while in case of BCE-loss, the heatmap is noticeably more diffuse and less focused on the true disease regions. This clearer focus on sickle-cell morphology helps explain why the triplet-loss model outperforms other losses.

## Training from scratch compared to transfer learning

To further validate the utility of transfer learning in our low resource settings, we trained the architectures - ResNet-50, DenseNet-121, EfficientNet-B0, and Baseline MobileNetV2 - from scratch (that is, with weights = none). Despite matching our fine-tuned models in capacity and training procedure, these scratch models consistently underperformed. On average, their training accuracy trailed only $\approx 5\%$, but their validation and test accuracies suffered a far greater drop, as summarized in Table 4. It can be seen that, they achieve only 40–50% validation and test accuracies, which is roughly half of their pretrained counterparts. These results underscore that, in data-scarce clinical settings, the representations learned by pre-training on large-scale natural images confer critical generalization advantages.

**Table 4:** Performance of scratch-trained models (random initialization) on balanced training dataset.

Network	Loss	Epoch	Batch size	Learn rate	Validation accuracy	Test accuracy	Precision	Recall	F-1 score
ResNet-50	Triplet	30	32	0.0001	0.490	0.460	0.490	0.440	0.460
DenseNet-121	Triplet	30	32	0.0001	0.480	0.460	0.480	0.430	0.450
EfficientNet-B0	Triplet	30	32	0.0001	0.480	0.450	0.450	0.450	0.440
ResNet-50	BCE	30	16	0.0001	0.480	0.420	0.440	0.400	0.420
DenseNet-121	BCE	30	16	0.0001	0.490	0.440	0.460	0.430	0.440
EfficientNet-B0	BCE	30	16	0.0001	0.460	0.420	0.440	0.420	0.430
ResNet-50	Focal	30	32	0.0001	0.440	0.430	0.440	0.420	0.430
DenseNet-121	Focal	30	32	0.0001	0.490	0.440	0.460	0.430	0.440
EfficientNet-B0	Focal	30	32	0.0001	0.410	0.280	0.280	0.250	0.260

Apart from these, we also evaluated a lighter-weight baseline, **MobileNetV2**, trained both with ImageNet pretraining and from scratch, under all three losses (Triplet, BCE, and Focal). This experiment was intended to assess whether model capacity alone was responsible for overfitting and to verify if our conclusions hold across a smaller, computationally efficient architecture. However, the MobileNetV2 models (both pretrained and scratch) consistently underperformed compared to the larger backbones, achieving test accuracies in the range of 70–80% as seen in Table  5. This indicates that while capacity reduction mitigates some overfitting, it does not compensate for the representational strength of deeper models such as ResNet-50 and DenseNet-121, especially when combined with Triplet loss. Hence, we retain these architectures for our final analysis, as they best capture the morphological nuances required for sickle-cell classification while maintaining interpretability via Grad-CAM.

**Table 5:** Performance of lightweight MobileNetV2 baseline under identical training conditions.

Network	Initialization	Loss	Validation accuracy	Test accuracy	Precision	Recall	F-1 Score	Learn rate
MobileNetV2	Pretrained	Triplet	0.810	0.790	0.800	0.770	0.785	0.0001
MobileNetV2	Pretrained	BCE	0.780	0.760	0.770	0.740	0.755	0.0001
MobileNetV2	Pretrained	Focal	0.740	0.720	0.730	0.710	0.720	0.0001
MobileNetV2	Scratch	Triplet	0.400	0.390	0.410	0.380	0.395	0.0001
MobileNetV2	Scratch	BCE	0.380	0.350	0.370	0.340	0.355	0.0001
MobileNetV2	Scratch	Focal	0.320	0.250	0.290	0.260	0.275	0.0001

## Experiments with unbalanced training dataset

Note that, in real-world applications, the training dataset often contains an imbalance in terms of a number of samples. To investigate the robustness of our models to such imbalanced conditions, we experiment by using the original training dataset with a significantly different number of samples in the two classes. Here, the dataset contains 1185 images in the‘Normal’class and 800 images in the‘Sickle’class. Table 6 shows a summary of the results.

**Table 6:** Results obtained using a KNN back-end classifier on unbalanced training dataset.

Network	Loss	Epoch	Batch size	Learn rate	Validation accuracy	Test accuracy	Precision	Recall	F-1 Score
ResNet-50	Triplet	30	32	0.0001	0.984	0.930	0.977	0.880	0.892
DenseNet-121	Triplet	30	32	0.0001	0.955	0.915	0.966	0.860	0.934
EfficientNet-B0	Triplet	30	32	0.0001	0.950	0.900	0.900	0.900	0.869
ResNet-50	BCE	30	16	0.0001	0.951	0.840	0.877	0.790	0.852
DenseNet-121	BCE	30	16	0.0001	0.961	0.875	0.924	0.860	0.877
EfficientNet-B0	BCE	30	16	0.0001	0.930	0.835	0.838	0.830	0.831
ResNet-50	Focal	30	32	0.0001	0.883	0.860	0.875	0.840	0.852
DenseNet-121	Focal	30	32	0.0001	0.980	0.885	0.914	0.850	0.854
EfficientNet-B0	Focal	30	32	0.0001	0.820	0.550	0.558	0.500	0.579

The shift in class distribution yielded noticeable differences in the results obtained. ResNet-50 performed the best with triplet loss achieving a test accuracy of 93.00 percent, followed by DenseNet-121 and EfficientNet-B0 with triplet loss achieving 91.50 percent and 90.00 percent test accuracies, respectively, making this loss function the best suitable choice once again for this task. BCE loss showed commendable performance as it exhibited a less pronounced decline in the test accuracies with every model. Every model when paired up with BCE loss, maintained the test accuracies as before, even after employing them on the unbalanced dataset. Focal loss, specifically designed to address class imbalance, yielded inconclusive results in our experiments. While DenseNet-121 and ResNet-50 trained with Focal loss slightly improved their test accuracy on the imbalanced dataset compared to the balanced data, EfficientNet-B0 under-performed yet again, yielding an accuracy of just 55.00 percent. This suggests that Focal Loss might be model-dependent in its effectiveness for imbalanced data.

## Conclusion

This study helps us investigate the application of DL models for SCD detection from digitized microscopic blood smears, addressing the challenge of limited and imbalanced datasets. We leveraged transfer-learning with three pre-trained DNN models (ResNet-50, DenseNet-121, and EfficientNet-B0) and evaluated them with triplet loss, BCE loss and focal loss functions. We then compared the performance of these with the performances of respective models trained from scratch. Obtained results highlighted the superiority of transfer-learning based approach.

Furthermore, to study the effectiveness of the proposed approaches in unbalanced conditions, the models were trained and tested on unbalanced datasets as well. The balanced dataset analysis revealed that DenseNet-121 with triplet loss achieved the highest performance. However, when considering the real-world scenario of imbalanced data, ResNet-50 with triplet loss demonstrated superior resilience. DL models’ feature extraction capabilities are validated by training a KNN classifier on the representations obtained from the final layer. This KNN classifier achieved excellent results, demonstrating the effectiveness of our models in learning discriminative features for SCD detection. Furthermore, Grad-CAM, an XAI technique, was implemented to increase the interpretability of the model predictions, fostering trust and facilitating potential adjustments if needed. We adopt Gradient-weighted Class Activation Mapping (Grad-CAM) to interrogate model decisions for two reasons. First, sickle-cell detection depends on localized shape cues; Grad-CAM produces class-discriminative heatmaps that allow clinicians to verify that the network attends to the expected curved, elongated erythrocytes rather than spurious artifacts (e.g., stains or illumination seams). Second, it is computationally light and architecture-agnostic, enabling a uniform comparison across all backbone-loss combinations on the same images, without introducing method-specific hyperparameters. While Grad-CAM is coarse and can be sensitive to architectural details, it is sufficient for our validation goal, ensuring that improved accuracy under triplet loss coincides with sharper focus on true sickle morphology. We acknowledge that alternatives such as Layer-wise Relevance Propagation or XRAI can yield finer-grained attributions. However, we consider integrating them as complementary analyses in future work. To conclude, in performance and interpretability, our results show that triplet loss consistently outperforms BCE and focal loss baselines, with Grad-CAM saliency maps confirming that the model attends more faithfully to sickled regions rather than spurious artifacts. This makes the proposed system practical for integration into portable diagnostic pipelines, where a technician could capture microscope images and instantly receive both a classification and an interpretable heatmap, enabling faster screening and referral in settings where advanced diagnostic facilities are unavailable. Notably, none of the prior SCD studies (surveyed in Table 1) adopt metric learning, in particular, triplet loss. They rely instead on conventional detection/segmentation/classification pipelines optimized with cross-entropy-family objectives. This gap motivates our approach: we learn a discriminative embedding space with triplet loss and then classify in that space, which proves robust in low-resource, class-imbalanced settings and translates into stronger accuracy in our experiments.

## Future work

For future work, multiple directions could be used to improve the robustness of our model for SCD detection. Firstly, while we used limited-size dataset and computing resources, expanding the dataset and utilizing more powerful computational resources could improve model accuracy and generalization capabilities. Access to a larger, more diverse dataset would provide the model with a wider range of cell morphologies, lighting conditions, and staining variations, likely enhancing its ability to identify SCD with high precision across various clinical settings.

Secondly, cross-dataset validation represents an area of potential improvement. Typically, models trained on a given dataset tend to under-perform when tested on a different dataset due to variations in imaging techniques, resolution, or sample preparation. Implementing cross-dataset validation could identify these generalization challenges and guide the development of more robust models. Recent advancements in cross-dataset testing for Facial Action Unit detection focus on methods to enhance model generalizability across diverse datasets. Techniques like domain adaptation^54^ and selective retraining are used to align feature distributions between datasets. For instance, Chu et al. introduced a Selective Transfer Machine (STM) that re-weights training samples based on their relevance to the test subject, thereby reducing dataset bias^55^.

## Data Availability

Data related to plots and results in the manuscript is available from corresponding author upon reasonable request. The sickle cell images and details are available at: https://rdr.ucl.ac.uk/articles/dataset/Digitized_Thin_Blood_Films_for_Sickle_Cell_Disease_Detection/12407567/1.

## Code availability

Code for dataset pre-processing and model implementation is available at https://github.com/jp2404/Sickle-Cell-Disease-Detection-.
